# Thermal management of epidermal electronic devices/skin system considering insensible sweating

**DOI:** 10.1038/s41598-018-32152-4

**Published:** 2018-09-20

**Authors:** Shuang Nie, Chenxi Zhang, Jizhou Song

**Affiliations:** Department of Engineering Mechanics, Soft Matter Research Center, and Key Laboratory of Soft Machines and Smart Devices of Zhejiang Province, Zhejiang University, Hangzhou, 310027 China

## Abstract

Thermal management of the system consisting of epidermal electronics devices (EEDs) and skin is critically important since a few degrees in temperature increase may induce thermal discomfort. In this paper, considering insensible sweating, a three-dimensional analytical thermal model, validated by finite element analysis, is developed to derive analytical expressions for the steady temperature distributions in the EED/skin system. The influences of various parameters including the thickness and thermal conductivity of the substrate in EEDs on the maximum skin temperature are investigated. The comfort analysis is then carried out based on the model to provide design guidelines for optimizing the geometric and material properties of EEDs to avoid the adverse thermal effects. These results pave the theoretical foundation for thermal management of EEDs/skin system in practical applications.

## Introduction

**Figure 1:**
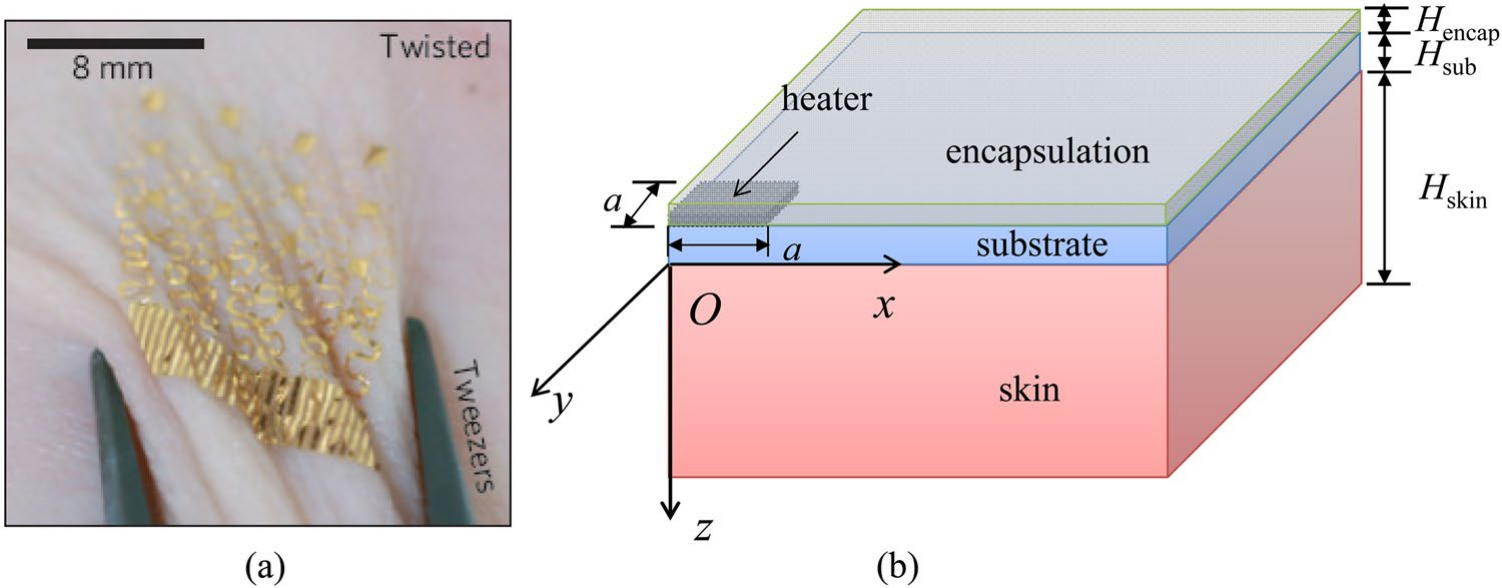
(**a**) An epidermal electronic device (EED) consisting of temperature sensors and heaters on a skin in a twisting motion. Reproduced with permission from ref.^3^. Copyright Nature Publishing Group. (**b**) Schematic diagram of the cross-sectional structure of the EED/skin system.

Epidermal electronic devices (EEDs) have attracted much attention recently due to their similar mechanical properties to those of skin, which give them capabilities to have a conformal contact with the skin even under complex deformations^1,2^. Figure 1(a) shows an EED consisting of temperature sensors and heaters on the skin under twist^3^. Various EEDs have been fabricated to show their potential in novel bio-integrated applications especially in the area of health monitoring^3–10^. Among these EEDs, many consist of heating components such as light-emitting diodes^7^ and heaters^5,10^. For the applications involving EEDs with heating components, thermal management of the EED/skin system is extremely important since the heating components may induce a temperature rise in the skin, which can cause thermal discomfort.

Considering skin effects on EED/skin system is more necessary for thermal management^11^. Many researchers have performed thermal studies of the EED/skin system in order to avoid the adverse thermal effects^12–14^. In these studies, the heat transfer in EEDs is modeled by the Fourier heat conduction equation

1 $$k{\nabla }^{2}T=\rho c\frac{\partial T}{\partial t}$$

with *t* as time, ${\nabla }^{2}$ as the Laplace operator, *T* as the temperature, *ρ* as the mass density, *c* as the heat capacity, and *k* as the thermal conductivity, while the heat transfer in the skin is modeled by the Pennes bioheat transfer equation^15^

2 $${k}_{skin}{\nabla }^{2}T-{\varpi }_{b}{\rho }_{b}{c}_{b}(T-{T}_{b})+{q}_{met}={\rho }_{skin}{c}_{skin}\frac{\partial T}{\partial t},$$

with *k*_*skin*_, *ρ*_*skin*_ and *c*_*skin*_ as the thermal conductivity, mass density and heat capacity of the skin, respectively, *ϖ*_*b*_ as the blood perfusion rate, *ρ*_*b*_ as the blood density, *c*_*b*_ as the heat capacity of blood, *T*_*b*_ as the blood temperature (same as the core body temperature), and *q*_*met*_ as the metabolic heat generation in the skin. The Pennes bioheat transfer equation regards the tissue as a homogeneous continuum material satisfying the Fourier heat conduction theory. It is rational to have the first term on the left-hand side of Eq. (2). The Pennes bioheat transfer equation accounts for the influences of blood perfustion and metabolism on the thermoregulation of skin via the second and third terms on the left-hand side of Eq. (2), respectively, but ignores the influence of sweating, which also contributes significantly to the thermoregulation of skin. This motivates us to develop a more accurate thermal model considering sweating for the EED/skin system to pave the theoretical foundation for thermal management.

It should be noted that there are two ways for sweating under normal conditions. One is insensible sweating with sweat evaporating before it is perceived as moisture on the skin surface when the ambient temperature is low. The other is sensible sweating with sweat flowing to the skin surface and then evaporating from the skin surface to decrease the skin temperature when the ambient temperature is high. The flow of sweat in the skin can be treated as a flow in a porous medium such that the influence of sweat can be described by modifying the Pennes bioheat transfer equation as^16,17^

3 $${k}_{skin}{\nabla }^{2}T-{\rho }_{s}{c}_{s}\bar{{\bf{V}}}\cdot \nabla T-{\varpi }_{b}{\rho }_{b}{c}_{b}(T-{T}_{b})+{q}_{met}+{q}_{s}={\rho }_{skin}{c}_{skin}\frac{\partial T}{\partial t},$$

where $\bar{{\bf{V}}}$ is sweat velocity in the duct, *ρ*_*s*_ and *c*_*s*_ are the mass density and heat capacity of the sweat, respectively, $-{\rho }_{s}{c}_{s}\bar{{\bf{V}}}\cdot \nabla T$ is the convective heat transfer of sweat with skin, and *q*_*s*_ is the heat released in the duct. For the EED/skin system, the multilayer feature combined with the coupled heat transfer equations in Eqs (1) and (3) for different layers complicates the problem and makes it hard to find analytical solutions especially for sensible sweating where the influences of sweat sensed as moisture on the skin surface and heat loss through evaporation of the sweat to the environment must be considered.

This paper aims to develop an analytical thermal model accounting for insensible sweating, where the sweat is not sensed as moisture on the skin surface, for the EED/skin system to derive analytical expressions for the steady temperature distributions in the system. The comfort analysis is then carried out based on the model to provide design guidelines to optimize the geometric and material properties of EEDs to avoid the adverse thermal effects. The paper is outlined as follows. The analytical thermal model considering insensible sweating for the EED/skin system is described in Sec. 2. The results and discussion are presented in Sec. 3. The conclusions are summarized in Sec. 4.

## Analytical Thermal Model for the EED/Skin System

Figure 1(b) shows a quarter of a typical multilayered EED/skin system by taking advantage of symmetry with the EED consisting of a heating component (e.g., heater) located at the interface between the compliant substrate and encapsulation. The coordinate system is established with the origin *O* at the substrate/skin interface, *x* pointing to the right, *y* pointing to the front and *z* pointing from the top to the bottom. The thickness of each layer is denoted by *H* with the subscripts encap, sub, skin denoting for the encapsulation layer, substrate and skin, respectively. The in-plane size of the heater is 2*a* × 2*a* × *H*_*heater*_ with *a* as the half-size and *H*_*heater*_ as the thickness of the heater. The bottom surface of the skin is set as the core body temperature *T*_*b*_. The top surface of the encapsulation layer has a convection boundary with the coefficient convection denoted by *h*. The heater, which is usually made of metal (e.g., gold), can be modeled as a planar heat source since its thickness (~50 nm) is much smaller than those (~10 μm) of encapsulation layer and substrate.

The steady temperatures *T*_*encap*_ and *T*_*sub*_ in the encapsulation layer and substrate satisfy the Fourier heat conduction equation in Eq. (1) while the steady temperature *T*_*skin*_ in the skin satisfies the bioheat transfer equation in Eq. (3) by excluding the time terms. The heat transfer equations for the encapsulation layer, substrate and skin can be written as the following system of nonhomogeneous equations

4 $$\{\begin{array}{l}{k}_{encap}(\frac{{\partial }^{2}T}{\partial {x}^{2}}+\frac{{\partial }^{2}T}{\partial {y}^{2}}+\frac{{\partial }^{2}T}{\partial {z}^{2}})=0\,-\,{H}_{encap}-\,{H}_{sub}\le z\le -\,{H}_{sub}\\ {k}_{sub}(\frac{{\partial }^{2}T}{\partial {x}^{2}}+\frac{{\partial }^{2}T}{\partial {y}^{2}}+\frac{{\partial }^{2}T}{\partial {z}^{2}})=0\,-{H}_{sub}\le z\le 0\\ {k}_{skin}(\frac{{\partial }^{2}T}{\partial {x}^{2}}+\frac{{\partial }^{2}T}{\partial {y}^{2}}+\frac{{\partial }^{2}T}{\partial {z}^{2}})-{\rho }_{s}{c}_{s}{V}_{z}\frac{\partial T}{\partial z}-{\varpi }_{b}{\rho }_{b}{c}_{b}(T-{T}_{b})+{q}_{met}+{q}_{s}=0\,\,{\rm{0}}\le z\le {H}_{skin}\end{array},$$

where *k*_*encap*_ *k*_*sub*_ and *k*_*skin*_ are the thermal conductivities of the encapsulation layer, substrate and skin, respectively, *V*_*z*_ is the sweat flow velocity along the *z* direction. Here the sweat flow along the in-plane directions is ignored since the duct to carry the sweat flow is mainly along the *z* direction.

The convection boundary on the top surface (*z* = −*H*_*encap*_ − *H*_*sub*_) of the encapsulation layer gives

5 $${-{k}_{encap}\frac{\partial T}{\partial z}|}_{z=-({H}_{encap}+{H}_{sub})}={h({T}_{\infty }-T)|}_{z=-({H}_{encap}+{H}_{sub})},$$

where *h* is coefficient of heat convection and *T*_∞_ is the ambient temperature. At the encapsulation/substrate interface (*z* = −*H*_*sub*_), the temperature is continuous and the heat flux satisfies the heat source condition, which yield

6 $${T|}_{z=-{{H}_{sub}}^{-}}={T|}_{z=-{{H}_{sub}}^{+}},$$

and

7 $$\{\begin{array}{ll}{-{k}_{sub}\frac{\partial T}{\partial z}|}_{z=-{{H}_{sub}}^{+}}+{{k}_{encap}\frac{\partial T}{\partial z}|}_{z=-{{H}_{sub}}^{-}}=0 & x\notin [0,\,a],\,y\notin [0,\,a]\\ {-{k}_{sub}\frac{\partial T}{\partial z}|}_{z=-{{H}_{sub}}^{+}}+{{k}_{encap}\frac{\partial T}{\partial z}|}_{z=-{{H}_{sub}}^{-}}=\frac{Q}{4{a}^{2}} & x\in [0,\,a],\,y\in [0,\,a],\end{array}$$

with *Q* as the heat generation power of the heater. At the substrate/skin interface (*z* = 0), both the temperature and heat flux are continuous, i.e.,

8 $${T|}_{z={0}^{-}}={T|}_{z={0}^{+}},\,-\,{k}_{sub}{\frac{\partial T}{\partial z}|}_{z={0}^{-}}=-\,{k}_{skin}{\frac{\partial T}{\partial z}|}_{z={0}^{+}}.$$

The constant core body temperature at the bottom surface of skin gives

9 $${T|}_{z={H}_{skin}}={T}_{b}.$$

The analytical solution for the heat transfer equations in Eq. (4) with boundary conditions in Eqs (5–9) can be obtained by the superposition method, i.e.,

10 $$T(x,\,y,\,z)=\theta (x,\,y,\,z)+{\rm{\Delta }}T(x,\,y,\,z).$$

Here *θ*(*x*, *y*, *z*) is the temperature satisfying Eqs (4–9) when the heater is not working (*Q* = 0), which only depends *z* and can be solved as

11 $$\{\begin{array}{lc}{\theta }_{encap}={A}_{1}z+{A}_{2}+{T}_{b} & -{H}_{encap}-{H}_{sub}\le z\le -\,{H}_{sub}\\ {\theta }_{sub}={B}_{1}z+{B}_{2}+{T}_{b} & -{H}_{sub}\le z\le 0\\ {\theta }_{skin}={C}_{1}{e}^{{\gamma }_{1}z}+{C}_{2}{e}^{{\gamma }_{2}z}+{T}_{b}+q & {\rm{0}}\le z\le {H}_{skin},\end{array}$$

where ${\gamma }_{1}=(\alpha +\sqrt{{\alpha }^{2}+4\beta })/2$ and ${\gamma }_{2}=(\alpha -\sqrt{{\alpha }^{2}+4\beta })/2$ with *α* = *ρ*_*s*_*c*_*s*_*V*_*z*_/*k*_*skin*_ and *β* = *ϖ*_*b*_*ρ*_*b*_*c*_*b*_*V*_*z*_/*k*_*skin*_, *q* = (*q*_*met*_ + *q*_*s*_)*k*_*skin*_/(*ϖ*_*b*_*ρ*_*b*_*c*_*b*_), and

12 $$\{\begin{array}{c}{A}_{1}\\ {A}_{2}\\ {B}_{1}\\ {B}_{2}\\ {C}_{1}\\ {C}_{2}\end{array}\}=\frac{1}{EF-{k}_{encap}{k}_{sub}hG}\{\begin{array}{c}{k}_{sub}{k}_{skin}h[\tau F+q({\gamma }_{2}-{\gamma }_{1})]\\ {k}_{sub}{k}_{skin}h[\tau F+q({\gamma }_{2}-{\gamma }_{1})]({H}_{encap}+{H}_{sub}+\frac{{k}_{encap}}{h})\\ {k}_{encap}{k}_{skin}h[\tau F+q({\gamma }_{2}-{\gamma }_{1})]\\ {k}_{encap}{k}_{sub}hG(\tau -q)+E({\gamma }_{2}-{\gamma }_{1})q+EFq\\ {k}_{encap}{k}_{sub}h{e}^{{\gamma }_{2}{H}_{skin}}\tau +(E{\gamma }_{2}-{k}_{encap}{k}_{sub}h)q\\ -{k}_{encap}{k}_{sub}h{e}^{{\gamma }_{1}{H}_{skin}}\tau -(E{\gamma }_{1}-{k}_{encap}{k}_{sub}h)q\end{array}\},$$

with *E* = *k*_*sub*_*k*_*skin*_*H*_*encap*_*h* + *k*_*encap*_*k*_*skin*_*H*_*sub*_*h* + *k*_*encap*_*k*_*sub*_*k*_*skin*_, $F={\gamma }_{1}{e}^{{\gamma }_{2}{H}_{skin}}-{\gamma }_{2}{e}^{{\gamma }_{1}{H}_{skin}}$, $G={e}^{{\gamma }_{2}{H}_{skin}}-{e}^{{\gamma }_{1}{H}_{skin}}$, *τ* = *T*_*b*_ − *T*_∞_ + *q*.

Δ*T* (*x*, *y*, *z*) is the temperature increase from the core body temperature due to the heat generation power of *Q* when the heater is working and it satisfies

13 $$\{\begin{array}{l}{k}_{encap}(\frac{{\partial }^{2}{\rm{\Delta }}T}{\partial {x}^{2}}+\frac{{\partial }^{2}{\rm{\Delta }}T}{\partial {y}^{2}}+\frac{{\partial }^{2}{\rm{\Delta }}T}{\partial {z}^{2}})=0\,-\,{H}_{encap}-\,{H}_{sub}\le z\le -\,{H}_{sub}\\ {k}_{sub}(\frac{{\partial }^{2}{\rm{\Delta }}T}{\partial {x}^{2}}+\frac{{\partial }^{2}{\rm{\Delta }}T}{\partial {y}^{2}}+\frac{{\partial }^{2}{\rm{\Delta }}T}{\partial {z}^{2}})=0\,-\,{H}_{sub}\le z\le 0\\ {k}_{skin}(\frac{{\partial }^{2}{\rm{\Delta }}T}{\partial {x}^{2}}+\frac{{\partial }^{2}{\rm{\Delta }}T}{\partial {y}^{2}}+\frac{{\partial }^{2}{\rm{\Delta }}T}{\partial {z}^{2}})-{\rho }_{s}{c}_{s}{V}_{z}\frac{\partial {\rm{\Delta }}T}{\partial z}-{\varpi }_{b}{\rho }_{b}{c}_{b}{\rm{\Delta }}T=0\,\,{\rm{0}}\le z\le {H}_{skin}\end{array},$$

and

14 $$-{k}_{encap}{\frac{\partial {\rm{\Delta }}T}{\partial z}|}_{z=-({H}_{encap}+{H}_{sub})}=0,$$

15 $${{\rm{\Delta }}T|}_{z=-{{H}_{sub}}^{-}}={{\rm{\Delta }}T|}_{z=-{{H}_{sub}}^{+}},$$

16 $$\{\begin{array}{ll}-{k}_{sub}{\frac{\partial {\rm{\Delta }}T}{\partial z}|}_{z=-{{H}_{sub}}^{+}}+{k}_{encap}{\frac{\partial {\rm{\Delta }}T}{\partial z}|}_{z=-{{H}_{sub}}^{-}}=0 & x\notin [0,a],\,y\notin [0,\,a]\\ -{k}_{sub}{\frac{\partial {\rm{\Delta }}T}{\partial z}|}_{z=-{{H}_{sub}}^{+}}+{k}_{encap}{\frac{\partial {\rm{\Delta }}T}{\partial z}|}_{z=-{{H}_{sub}}^{-}}=\frac{Q}{4{a}^{2}} & x\in [0,a],\,y\in [0,\,a],\end{array}$$

17 $${{\rm{\Delta }}T|}_{z={0}^{-}}={{\rm{\Delta }}T|}_{z={0}^{+}},\,-\,{k}_{sub}{\frac{\partial {\rm{\Delta }}T}{\partial z}|}_{z={0}^{-}}=-\,{k}_{skin}{\frac{\partial {\rm{\Delta }}T}{\partial z}|}_{z={0}^{+}},$$

18 $${{\rm{\Delta }}T|}_{z={H}_{skin}}=0.$$

The last two terms in the third equation of Eq. (4) is not included in Eq. (13) because they have already been included in the equations solving for *θ*(*x*, *y*, *z*), i.e., Δ*T*(*x*, *y*, *z*) only needs to satisfy the homogeneous equations. The Fourier transform ${\rm{\Delta }}\overline{T}(\xi ,\,\eta ,\,z)={\int }_{-\infty }^{\infty }{\int }_{-\infty }^{\infty }{\rm{\Delta }}T(x,\,y,\,z){e}^{-i(\xi x+\eta y)}dxdy$ of Eqs (13–18) yields

19 $$\{\begin{array}{l}\frac{{\partial }^{2}{\rm{\Delta }}\bar{T}}{\partial {z}^{2}}-({\xi }^{2}+{\eta }^{2}){\rm{\Delta }}\bar{T}=0\,-\,{H}_{encap}-\,{H}_{sub}\le z\le -{H}_{sub}\\ \frac{{\partial }^{2}{\rm{\Delta }}\bar{T}}{\partial {z}^{2}}-({\xi }^{2}+{\eta }^{2}){\rm{\Delta }}\bar{T}=0\,-{H}_{sub}\le z\le 0\\ \frac{{\partial }^{2}{\rm{\Delta }}\bar{T}}{\partial {z}^{2}}-\frac{{\rho }_{s}{c}_{s}{V}_{z}}{{k}_{skin}}\frac{\partial {\rm{\Delta }}\bar{T}}{\partial z}-(\frac{{\varpi }_{b}{\rho }_{b}{c}_{b}}{{k}_{3}}+{\xi }^{2}+{\eta }^{2}){\rm{\Delta }}\bar{T}=0\,\,{\rm{0}}\le z\le \,{H}_{skin}\end{array},$$

and

20 $$-{k}_{encap}{\frac{\partial {\rm{\Delta }}\bar{T}}{\partial z}|}_{z=-({H}_{encap}+{H}_{sub})}=0,$$

21 $${{\rm{\Delta }}\bar{T}|}_{z=-{{H}_{sub}}^{-}}={{\rm{\Delta }}\bar{T}|}_{z=-{{H}_{sub}}^{+}},$$

22 $$-{k}_{sub}{\frac{\partial {\rm{\Delta }}\bar{T}}{\partial z}|}_{z=-{{H}_{sub}}^{+}}+{k}_{encap}{\frac{\partial {\rm{\Delta }}\bar{T}}{\partial z}|}_{z=-{{H}_{sub}}^{-}}=\frac{\sin (\xi a)\sin (\eta a)Q}{\xi \eta {a}^{2}},$$

23 $${{\rm{\Delta }}\bar{T}|}_{z={0}^{-}}={{\rm{\Delta }}\bar{T}|}_{z={0}^{+}},\,-\,{k}_{sub}{\frac{\partial {\rm{\Delta }}\bar{T}}{\partial z}|}_{z={0}^{-}}=-\,{k}_{skin}{\frac{\partial {\rm{\Delta }}\bar{T}}{\partial z}|}_{z={0}^{+}},$$

24 $${{\rm{\Delta }}\bar{T}|}_{z={H}_{skin}}=0.$$

which can be solved analytically as

25 $$\{\begin{array}{cccc}{\rm{\Delta }}{T}_{encap} & = & {\bar{A}}_{1}{e}^{\lambda z}+{\bar{A}}_{2}{e}^{-\lambda z} & -{H}_{encap}-{H}_{sub}\le z\le -\,{H}_{sub}\\ {\rm{\Delta }}{T}_{sub} & = & {\bar{B}}_{1}{e}^{\lambda z}+{\bar{B}}_{2}{e}^{-\lambda z} & -{H}_{sub}\le z\le 0\\ {\rm{\Delta }}{T}_{skin} & = & {\bar{C}}_{1}{e}^{{\zeta }_{1}z}+{\bar{C}}_{2}{e}^{{\zeta }_{2}z} & {\rm{0}}\le z\le {H}_{skin}.\end{array}$$

Here

26 $$\{\begin{array}{c}{\bar{A}}_{1}\\ {\bar{A}}_{2}\end{array}\}=\frac{\bar{G}[{k}_{sub}+({k}_{encap}-{k}_{sub})\lambda ]}{{k}_{encap}}\{\begin{array}{c}\frac{1}{1-{e}^{-\lambda (2{H}_{encap}+{H}_{sub})}}\\ \frac{1}{{e}^{\lambda {H}_{sub}}({e}^{2\lambda {H}_{encap}}-1)}\end{array}\},$$

and

27 $$\{\begin{array}{c}{\bar{B}}_{1}\\ {\bar{B}}_{2}\\ {\bar{C}}_{1}\\ {\bar{C}}_{2}\end{array}\}=\frac{\bar{G}{[{k}_{skin}{\zeta }_{1}{\zeta }_{2}\bar{F}-{k}_{sub}\lambda \tanh ({e}^{\lambda {H}_{sub}})\bar{E}]}^{-1}}{({e}^{-\lambda {H}_{sub}}+{e}^{\lambda {H}_{sub}})}\{\begin{array}{c}{k}_{sub}\lambda \bar{E}+{k}_{skin}{\zeta }_{2}{\zeta }_{1}\bar{F}\\ {k}_{sub}\lambda \bar{E}-{k}_{skin}{\zeta }_{2}{\zeta }_{1}\bar{F}\\ -2\lambda {k}_{sub}{\zeta }_{2}{e}^{{\zeta }_{2}{H}_{skin}}\\ 2\lambda {k}_{sub}{\zeta }_{1}{e}^{{\zeta }_{1}{H}_{skin}}\end{array}\},$$

with $$\bar{G}=\sin (\xi a)\sin (\eta a)Q/[\xi \eta ({k}_{encap}-{k}_{sub}){a}^{2}{\lambda }^{2}]$$, $$\bar{E}={\zeta }_{1}{e}^{{\zeta }_{1}{H}_{skin}}-{\zeta }_{2}{e}^{{\zeta }_{2}{H}_{skin}}$$, $$\bar{F}={e}^{{\zeta }_{1}{H}_{skin}}-{e}^{{\zeta }_{2}{H}_{skin}}$$, $$\lambda =\sqrt{{\xi }^{2}+{\eta }^{2}}$$, $${\zeta }_{1}=(\bar{\alpha }+\sqrt{{\bar{\alpha }}^{2}+4\bar{\beta }})/2$$, $${\zeta }_{2}=(\bar{\alpha }-\sqrt{{\bar{\alpha }}^{2}+4\bar{\beta }})/2$$, $$\bar{\alpha }={\rho }_{s}{c}_{s}{V}_{z}/{k}_{skin}$$ and $$\bar{\beta }={\varpi }_{b}{\rho }_{b}{c}_{b}{V}_{z}/{k}_{skin}+{\xi }^{2}+{\eta }^{2}$$.

The inverse Fourier transform ${\rm{\Delta }}T(x,y,z)=\frac{1}{4{\pi }^{2}}{\int }_{-\infty }^{\infty }{\int }_{-\infty }^{\infty }{\rm{\Delta }}\overline{T}(\xi ,\eta ,z){e}^{i(\xi x+\eta y)}d\xi d\eta$ then gives the temperature increase in the system. For example, the temperature increase at the substrate/skin interface is obtained as

28 $${\rm{\Delta }}T(x,\,y,\,z=0)=\frac{1}{4{\pi }^{2}}{\int }_{-\infty }^{\infty }{\int }_{-\infty }^{\infty }({\bar{C}}_{1}+{\bar{C}}_{2}){e}^{i(\xi x+\eta y)}d\xi d\eta .$$

The maximum temperature increase in the skin occurs at (0, 0, 0) on the substrate/skin interface and can be given by

29 $${\rm{\Delta }}{T}_{skin}^{\max }=\frac{1}{4{\pi }^{2}}{\int }_{-\infty }^{\infty }{\int }_{-\infty }^{\infty }({\bar{C}}_{1}+{\bar{C}}_{2})\,d\xi d\eta .$$

The maximum skin temperature can then be obtained as

30 $${T}_{skin}^{\max }={\theta }_{skin}^{\max }+{\rm{\Delta }}{T}_{skin}^{\max }={C}_{1}+{C}_{2}+{T}_{b}+q+\frac{1}{4{\pi }^{2}}{\int }_{-\infty }^{\infty }{\int }_{-\infty }^{\infty }({\bar{C}}_{1}+{\bar{C}}_{2})\,d\xi d\eta .$$

which will be adopted to perform comfort analysis in Sec. 3.

## Results and Discussion

The material parameters of skin are listed in Table 1. The thermal conductivity and thickness of skin are 0.187 W/(m·K) and 6 mm, respectively^18^. The specific heat and density of blood are 3770 J/(kg·K) and 1060 kg/m^3^, respectively^18^. The blood perfusion rate is usually within the range between 0 and 0.1 ml Blood/ml tissue/s, and it is taken as 0.038 ml Blood/ml tissue/s in the calculations below^17^. The specific heat and density of sweat are 4200 J/(kg·K) and 1000 kg/m^3^, respectively^16^. The metabolic heat generation is 368.1 W/m^3^ ^18^. The blood temperature and core body temperature are 37 °C^17–19^.

**Table 1:** Material parameters of skin.

Parameters	Value
Skin thermal conductivity (W/m·K)	0.187
Blood specific heat (J/kg·K)	3770
Blood density (kg/m^3^)	1060
Blood perfusion rate (ml Blood/ml tissue/s)	0–0.1
Sweat specific heat (J/kg·K)	4200
Sweat density (kg/m^3^)	1000
Metabolic heat generation (W/m^3^)	368.1
Skin thickness (mm)	6
Arterial blood temperature (^o^C)	37
Core body temperature (^o^C)	37

A three dimensional finite element model is established in COMSOL to investigate the temperature distributions in the EED/skin system and to validate the analytical model developed in Sec. 2. Because Eq. (3) is more complicated than the general bioheat transfer equation, the module of “Coefficient form PDE” is used to solve the second order partial differential equation (PDE). This module provides a general interface for specifying and solving many well-known PDEs. The in-plane dimension of the system is set as 4 mm, which is large enough to eliminate the boundary influences. The top surface has a natural convection boundary with the heat convection coefficient *h* as 7 W/(m^2^·K). The temperature at the bottom surface is set as the body temperature 37 °C. The heater with the half size of *a* is located at the encapsulation/substrate interface and has the heat generation power of *Q*. If not specified, a is 150 μm and *Q* is 4 mW. The thickness and thermal conductivity are set to be 7 μm and 0.2 W/(m·K) for the encapsulation, and 1 mm and 0.6 W/(m·K) for the substrate, which are similar as those in flexible LEDs^20^.

**Figure 2:**
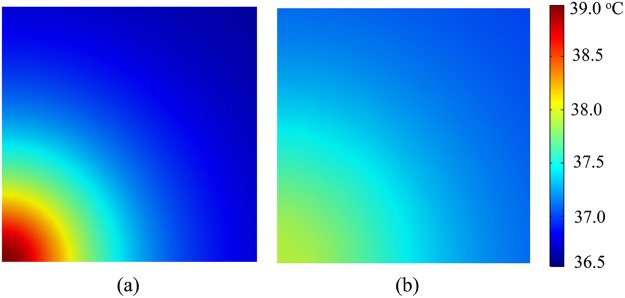
The temperature distributions at the substrate/skin interface from FEA (**a**) without and (**b**) with the influences of insensible sweating.

Figure 2 show the influences of insensible sweating on temperature distributions at the substrate/skin interface from finite element analysis (FEA). It is shown that the maximum temperature occurs at the center of heater, which gives the maximum skin temperature, and decreases as the distance to the center increases. When the insensible sweating is ignored, i.e., the sweat velocity is set to be zero, the maximum skin temperature can reach 39 °C. When the insensible sweating is accounted with the sweat velocity of 0.3 mm/s (only along *z* axis)^16^, the maximum skin temperature drops to 37.9 °C. These results clearly indicate that the influence of insensible sweating should be accounted in the temperature calculations for the EED/skin system.

**Figure 3:**
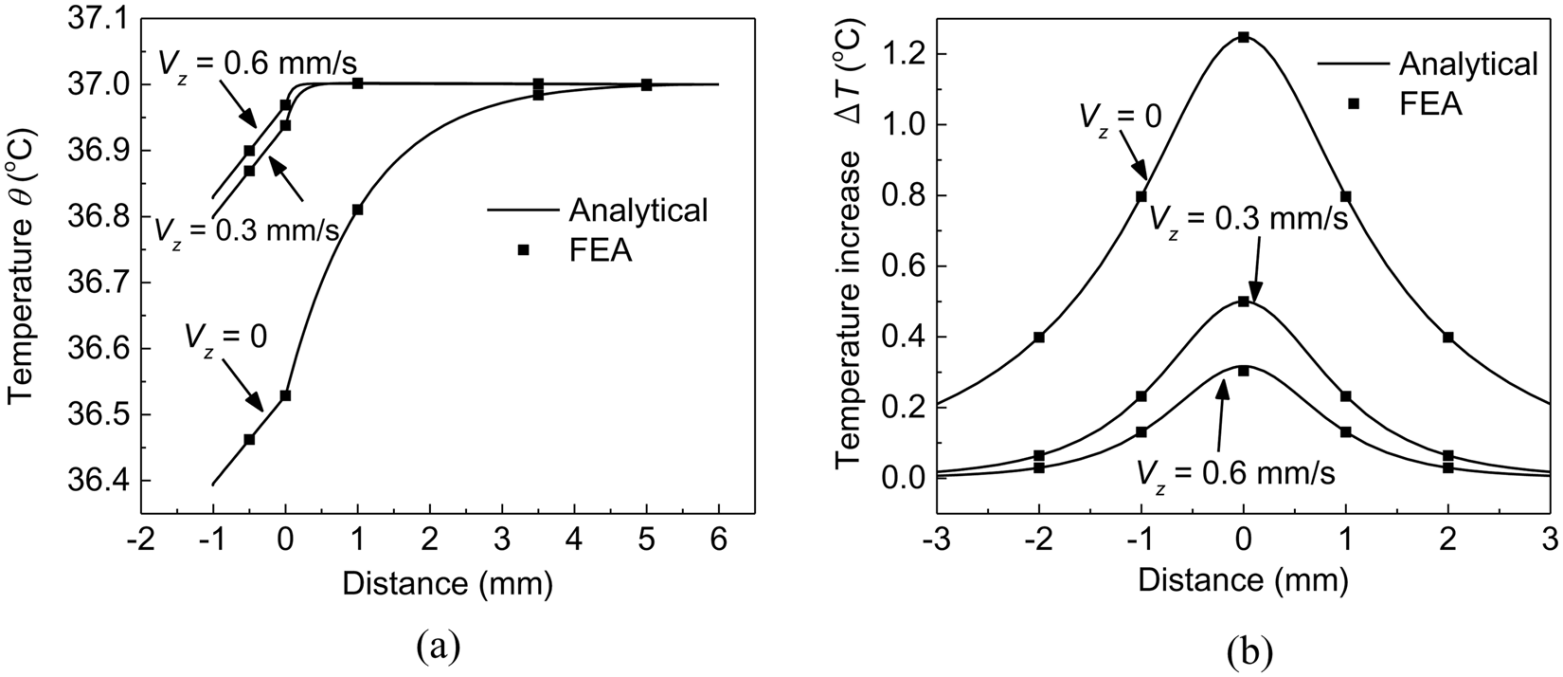
The effect of the sweat velocity on temperature distributions at the substrate/skin interface. (**a**) The distribution of temperature *θ* along the thickness direction (*z* axis) when the heater is not working; (**b**) The distribution of temperature increase Δ*T* along the in-plane direction (*x* axis) on the substrate/skin interface.

Based on the analytical model, the temperature *T* in the system can be obtained by superposing the temperature *θ* when the heater is not working and the temperature increase Δ*T* due to the heat generation power *Q* when the heater is working, i.e., *T* = *θ* + Δ*T*. Figure 3(a) shows the distribution of temperature *θ* along the *z* axis with and without considering the insensible sweating at the substrate/skin interface. The solid lines are obtained from analytical predictions in Eq. (11) while the solid dots are from FEA. The good agreement between the analytical predictions and FEA validates the analytical model. It is shown that the sweating increases *θ*. For example, as the sweat velocity increases from 0 to 0.6 mm/s, the temperature *θ* at the substrate/skin interface increases from 36.52 °C to 36.96 °C. The increase of *θ* with sweat velocity is due to the heat *q*_*s*_ released in the duct as well as the convective heat transfer, which yields the increase of *θ* when the sweat flows from the gland to the skin surface. Figure 3(b) shows the distribution of temperature increase Δ*T* along the *x* axis with and without considering the insensible sweating at the substrate/skin interface. The analytical predictions again agree well with FEA, which indicates the validity of the analytical model. Different from Fig. 3(a) for *θ*, the sweating decreases Δ*T*. For example, as the sweat velocity increases from 0 to 0.6 mm/s, the maximum Δ*T* decreases from 1.25 °C to 0.3 °C. The decrease of Δ*T* with sweat velocity is due to the convective heat transfer of sweat with skin (i.e., $-{\rho }_{s}{c}_{s}\bar{{\bf{V}}}\cdot \nabla T$), which dissipates more heat from the heater into the skin and yields the decrease of Δ*T*. The contrary influences of sweating on *θ* and Δ*T* may yield various outcomes of the maximum skin temperature depending on the material properties, geometric parameters of EEDs, and the loading parameters. For EEDs in this case, the sweating decreases the maximum skin temperature from 39 °C to 37.9 °C.

**Figure 4:**
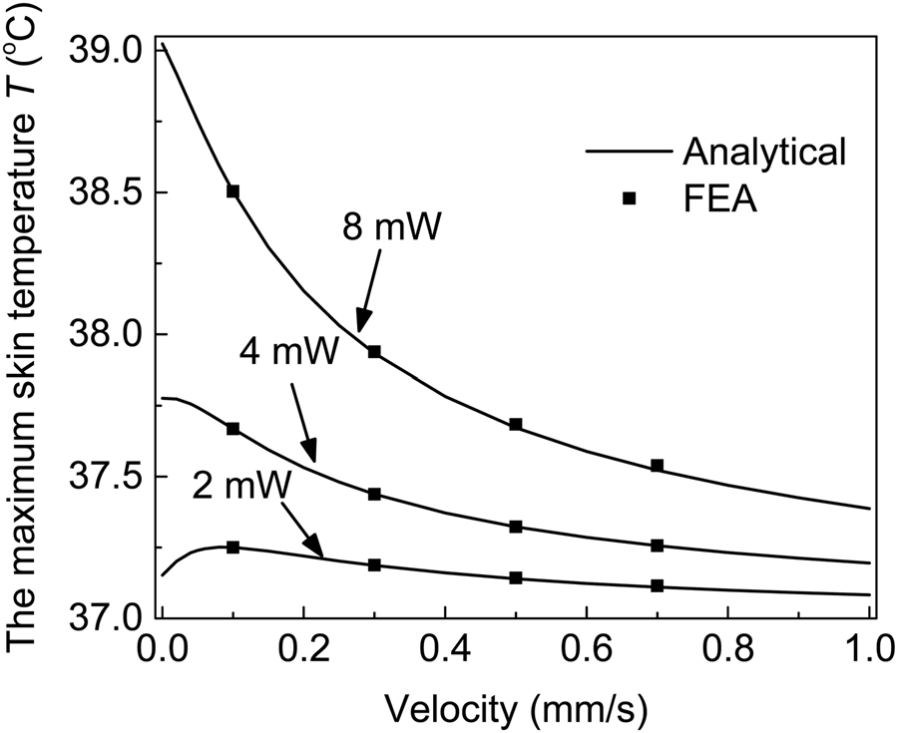
The maximum skin temperature versus the sweat velocity under various heat generation powers.

Figure 4 shows the maximum skin temperature versus the sweat velocity under various heat generation powers. The analytical predictions, denoted by solid line, from Eq. (30) agree well with FEA, denoted by solid dots. According to Eq. (10), the summation of *θ* and *ΔT* gives *T*. Due to the contrary influences of sweating on *θ* and Δ*T*, the influence of sweating on the maximum skin temperature also varies. When the heat generation power is small, the sweating increases the maximum skin temperature slightly at the beginning and then decreases the maximum skin temperature eventually with the increase of the sweat velocity. For example, for a small heat generation power of 2 mW, the maximum skin temperature increases slightly from 37.15 °C to 37.25 °C when the sweat velocity increases from 0 to 0.08 mm/s, and then decreases to 37.08 °C when the sweat velocity further increases to 1.0 mm/s. When the heat generation power is large, the maximum skin temperature decreases monotonously with the increase of the sweat velocity. For example, for a large heat generation power of 8 mW, the maximum skin temperature decreases monotonously from 39.02 °C to 37.20 °C with the increase of sweat velocity from 0 to 1.0 mm/s.

**Figure 5:**
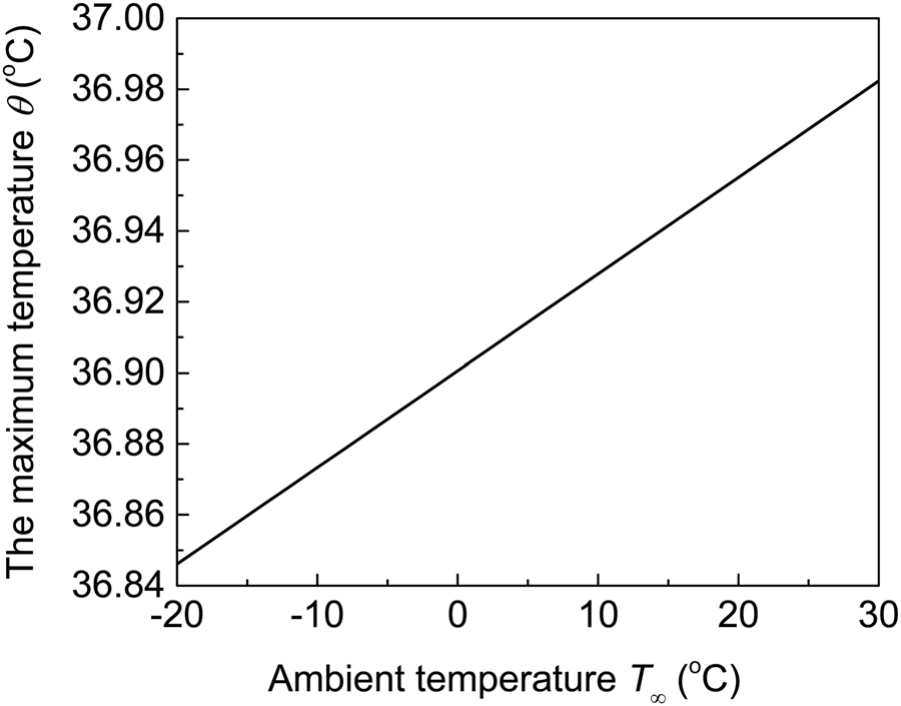
The effect of ambient temperature on the temperature *θ* at the substrate/skin interface.

The environment conditions (e.g. winter v.s. summer), which have been considered through the ambient temperature *T*_∞_ in the convection boundary on the top surface, may have influences on the maximum skin temperature. It should be noted that the ambient temperature affects the skin temperature only through *θ* but not the temperature increase Δ*T*. Figure 5 shows the influence of ambient temperature on *θ* at the substrate/skin interface, which is equivalent to the influence of ambient temperature on the maximum skin temperature. It is shown that the temperature *θ* at the substrate/skin interface increases linearly with the increase of ambient temperature. The influence of ambient temperature is negligible since the temperature *θ* at the substrate/skin interface (or the maximum skin temperature) only increases 0.13 °C when the ambient temperature increases from −20 °C to 30 °C. It should be noted that the above conclusion is only good for insensible sweating and the influence of ambient temperature may become noticeable when sensible sweating occurs under a high ambient temperature.

**Figure 6:**
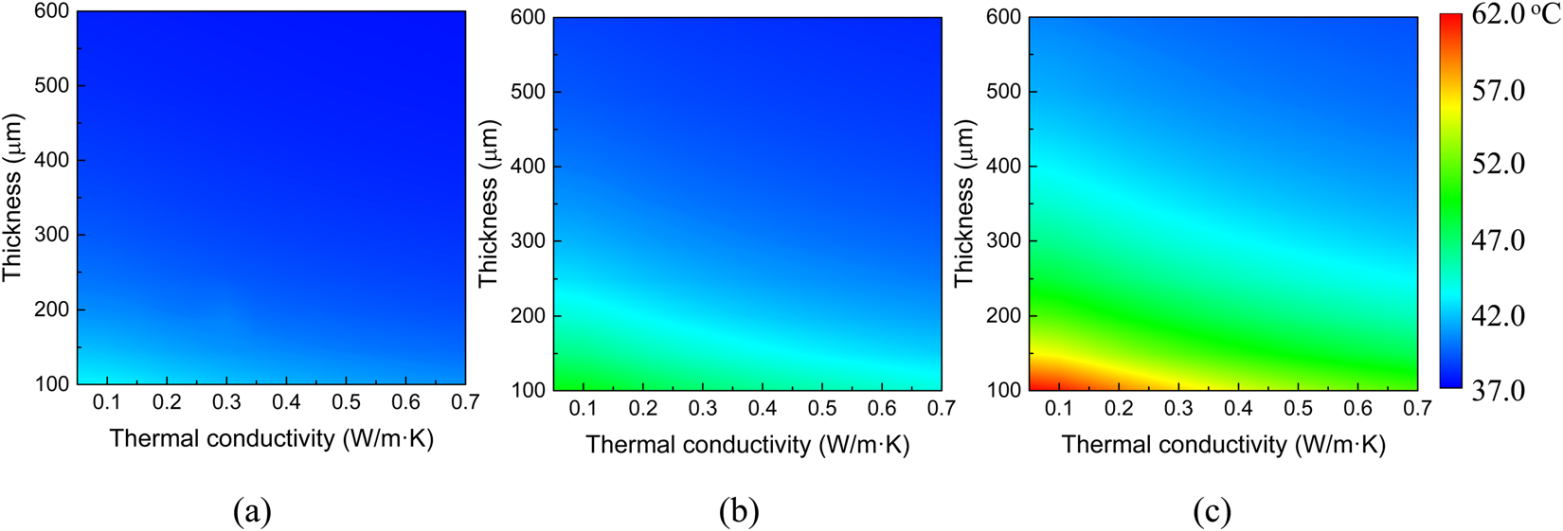
The effects of substrate thickness and thermal conductivity on the maximum skin temperature under the heat generation of (**a**) 2 mW, (**b**) 4 mW and (**c**) 8 mW. The sweat velocity is set as 0.3 mm/s.

The influences of the thickness and thermal conductivity of the substrate on the maximum skin temperature are shown in Fig. 6 with the sweat velocity of 0.3 mm/s. As the thermal conductivity of the substrate increases, the maximum skin temperature decreases. The maximum skin temperature decrease along with the larger thermal conductivity is due to the size effect of heater. For a large heater, the heat transfer can be approximated as one-dimensional. A higher thermal conductivity of the substrate results in more heat conducted to the skin, thus increases the maximum skin temperature. However, for a small heater (e.g., 150 μm), which is the case in the present work, the heat transfer is three-dimensional. A higher thermal conductivity of the substrate results in more heat conducted along the in-plane directions instead to the skin, thus decreases the maximum skin temperature. As the substrate thickness increases, the maximum skin temperature decreases since a thick substrate may prevent more heat to dissipate from the heat source to the skin. These results are very useful to design EEDs for comfort analysis to avoid the adverse thermal effects.

**Figure 7:**
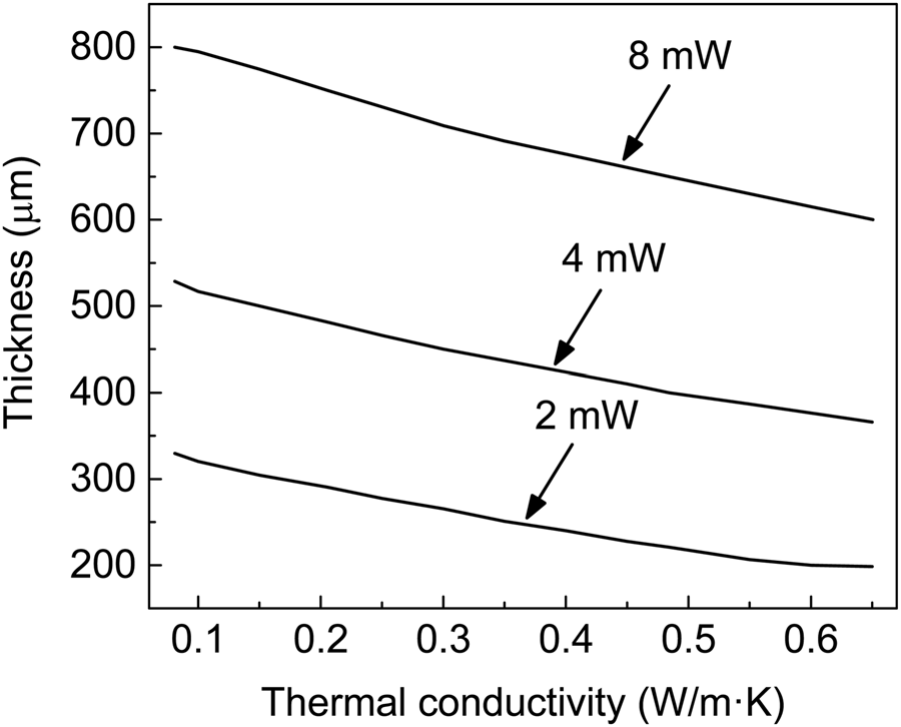
The critical substrate thickness and thermal conductivity to avoid thermal discomfort under various heat generation powers. The sweat velocity is set as 0.3 mm/s.

Zhu and Lu^21^ pointed out that the critical temperature 39 °C of thermal pain which can be felt by human is adopted here to perform comfort analysis. The thermal comfort region is defined as the range of material and geometric parameters giving the maximum skin temperature lower than 39 °C, while the thermal discomfort region is defined as the range of material and geometric parameters giving the maximum skin temperature higher than 39 °C. Figure 7 shows the critical substrate thickness and thermal conductivity to avoid thermal discomfort under various heat generation powers. The region above the curve, which has a lower temperature than the critical temperature, gives the thermal comfort region, while the region below the curve, which has a higher temperature than the critical temperature, gives the thermal discomfort region. These results provide design guidelines to select substrate thickness and thermal conductivity to avoid thermal discomfort.

## Conclusions

An analytical thermal model accounting for insensible sweating for the EED/skin system is established to derive analytical expressions for the steady temperature distributions. The analytical predictions agree well with finite element analysis. The influences of various parameters including the thickness and thermal conductivity of substrate in EEDs on the maximum skin temperature are investigated. The comfort analysis is then carried out based on the developed model and design guidelines on selecting the substrate thickness and thermal conductivity to avoid thermal discomfort are provided. These results pave the theoretical foundation for thermal management of EEDs/skin system considering insensible sweating.

## Data Availability

All data are available within the article or available from the authors upon reasonable request.
